# MT1JP-mediated miR-24-3p/BCL2L2 axis promotes Lenvatinib resistance in hepatocellular carcinoma cells by inhibiting apoptosis

**DOI:** 10.1007/s13402-021-00605-0

**Published:** 2021-05-11

**Authors:** Ting Yu, Jiajian Yu, Lu Lu, Yize Zhang, Yadong Zhou, Yong Zhou, Fengling Huang, Lu Sun, Zhixian Guo, Guojun Hou, Zihui Dong, Bibo Wang

**Affiliations:** 1 Department of Hepatobiliary, Fuling Central Hospital of Chongqing City, Chongqing, China; 2 Department of Medical Oncology, Jinling Hospital, School of Medicine, Nanjing University, Nanjing, China; 3 Precision Medicine Center, Gene Hospital of Henan Province, The First Affiliated Hospital of Zhengzhou University, Zhengzhou, China; 4 Department of Radiology, Fuling Central Hospital of Chongqing City, Chongqing, China; 5 Department of Infectious Diseases, The First Affiliated Hospital of Zhengzhou University, Zhengzhou, China; 6 The Third Department of Hepatic Surgery, Eastern Hepatobiliary Surgery Hospital, Second Military Medical University, Shanghai, China

**Keywords:** Hepatocellular carcinoma, Lenvatinib, Sorafenib, Resistance, Apoptosis

## Abstract

**Purpose:**

Lenvatinib is a long-awaited alternative to Sorafenib for first-line targeted therapy of patients with advanced hepatocellular carcinoma (HCC). However, resistance to Lenvatinib results in tumor progression and has become a major obstacle to improving the prognosis of HCC patients. Exploring the mechanisms underlying Lenvatinib resistance is considered essential for the treatment of advanced HCC.

**Methods:**

Lenvatinib resistant HCC (LR-HCC) cells were generated and potential long non-coding RNAs (Lnc-RNAs) upregulated in LR-HCC cells were identified by RNA sequencing. The effects of upregulated Lnc-RNAs were evaluated in vitro in cell models and in vivo in experimental animals using quantitative cell viability and apoptosis assays.

**Results:**

We found that Lnc-RNA MT1JP (MT1JP) was upregulated in LR-HCC cells and inhibited the apoptosis signaling pathway. In addition, we found that sponging of microRNA-24-3p by MT1JP released Bcl-2 like 2 (BCL2L2), an anti-apoptotic protein, thereby forming a positive-feedback loop. The role of this feedback loop was validated using rescue assays. Additionally, we found that upregulation of MT1JP and BCL2L2 impaired the sensitivity of HCC cells to Lenvatinib both vitro and vivo.

**Conclusions:**

Our results suggest a novel molecular feedback loop between MT1JP and apoptosis signaling in Lenvatinib sensitive HCC cells.

**Supplementary Information:**

The online version contains supplementary material available at 10.1007/s13402-021-00605-0.

## Introduction

Hepatocellular carcinoma (HCC) is one of the most frequent and aggressive cancers and the third leading cause of cancer-related death [1]. Approximately 700,000 new patients are diagnosed with HCC, annually. The development of HCC is tightly linked to sustained inflammation activated by either Hepatitis B virus (HBV) or Hepatitis C virus (HCV), alcohol abuse and/or metabolic disorders [2]. Despite clinical improvements that have been made, HCC patients are usually diagnosed with advanced stage disease. In addition, they frequently exhibit recurrences, resulting in five-year survival rates of < 20% [3]. Previous work has shown that hepatocellular carcinogenesis is a complex multi-step process, including genetic, epigenetic and cellular signaling alterations. As a result, HCCs extensively display heterogeneous molecular signatures [4]. As yet, however, our understanding of the molecular mechanisms underlying hepatocarcinogenesis is far from complete.

Since 2008, only Sorafenib, a tyrosine kinase inhibitor (TKI), has been approved by the food and drug administration (FDA) as first-line drug for unresectable HCC [5]. Although Regorafenib and Nivolumab were approved by the FDA as second-line treatments for Sorafenib-resistant HCC [6, 7], no other novel first-line drug was available for HCC until Lenvatinib (e7080), another TKI, was presented. In a randomized phase III clinical trial, it was found that Lenvatinib exhibited a non-inferior survival benefit compared to Sorafenib for untreated advanced HCC [8]. Lenvatinib has also been approved by the FDA for first-line treatment of HCC in August 2018.

Lenvatinib is an oral multi-kinase inhibitor that targets VEGF receptors, FGF receptors, PDGF receptor α, RET and KIT to suppress their downstream signaling pathways, including targets shared by Sorafenib [9, 10]. The clinical efficacy of Lenvatinib is well established now and has been found to extend HCC patient survival by 13.6 months, while for Sorafenib this was 12.3 months [8, 11]. This modest survival benefit may, however, be lost due to drug resistance, as has been observed for Sorafenib [12]. Lenvatinib primarily affects angiogenesis, and additional studies implied that orally administered Lenvatinib exhibited anti-angiogenic activity in thyroid cancer, lung cancer and HCC [8, 10, 13]. In addition, anti-proliferative effects of Lenvatinib have been observed in different cancer cell types, both in vitro and in vivo [14, 15]. Also, apoptosis-related caspase-3 and caspase-9 expression levels were found to be upregulated by Lenvatinib [16] and apoptotic rates to be increased in a Lenvatinib dose-dependent manner [15]. However, the mechanisms underlying Lenvatinib resistance (LR) are complicated and, as yet, largely unknown. Further investigations on the molecular basis of Lenvatinib resistance may shed light on the identification of new molecular targets to overcome this resistance.

Here, we generated two LR-HCC cell lines and performed RNA sequencing in these cell lines to systematically evaluate factors driving Lenvatinib resistance. We identified Lnc-MT1JP (MT1JP) as a critical driver of Lenvatinib resistance in HCC. In addition, we found that MT1JP functions as a molecular sponge of miR-24-3p to inhibit apoptosis through the anti-apoptotic protein BCL2 Like 2 (BCL2L2). Our findings indicate that the MT1JP/miR-24-3p/BCL2L2 axis contributes to resistance of HCC cells to Lenvatinib and that targeting MT1JP-induced apoptosis may be a promising strategy to overcome TKI resistance in human HCC.

## Materials and methods

### Cell culture and transfection

HCC cell lines SMMC-7721 and Huh7 were purchased from the Shanghai Institute of Cell Biology, Chinese Academy of Sciences. Both cell lines were maintained in DMEM supplemented with 10% fetal bovine serum and incubated at 37 °C in 5% CO_2_. MT1JP overexpression plasmid (pcDNA-MT1JP), MT1JP siRNAs, miR-24-3p mimics and miR-24-3p inhibitor, BCL2L2 overexpression plasmid, BCL2L2 siRNAs as well as negative control oligos were synthesized by GenePharm (Shanghai, China). For transfection, SMMC-7721 and Huh7 cells at 60%–80% confluence were transfected with these plasmids using Lipofectamine 2000 (Invitrogen) according to the manufacturer’s protocols.

### Establishment of Lenvatinib-resistant cell lines

To determine the IC_50_ of SMMC-7721 and Huh7 cells to Lenvatinib treatment, the cells were incubated with different concentrations of Lenvatinib in 96-well plates, followed by cell viability measurements 3 days later. In order to establish Lenvatinib-resistant cell lines, SMMC-7721 and Huh7 cells were incubated with Lenvatinib (Selleck, S1164) at a concentration just below their IC_50_, and increased by 0.2 μM/L per week for 6 to 7 months. Two Lenvatinib-resistant cell lines were obtained, termed 7721-LR and Huh7-LR. These two Lenvatinib-resistant cell lines were maintained by continued culture in the presence of Lenvatinib.

### qRT-PCR assay

Total RNA was isolated using Trizol reagent (Invitrogen) and reverse transcribed to cDNA using a TaqMan reverse transcription Kit (Applied Biosystems Life Technologies). qRT-PCR was performed using a SYBR Green PCR Kit (Roche) on an ABI Prism 7300 Sequence Detection System (Applied Biosystems). The primers used are listed in Supplementary Table 1.

### Western blotting

Total proteins were extracted using RIPA lysis buffer, and protein concentrations were determined using a BCA protein assay kit (Beyotime Biotechnology). The proteins (40 μg) were separated on 12% SDS-polyacrylamide gels and transferred to PVDF membranes (Millipore). The membranes were incubated with the following primary antibodies: anti-Caspase-3 (CST #9662), anti-Cleaved Caspase-3 (CST #9661), anti-PARP (CST #9532), anti-Cleaved PARP (CST #9548), anti-BCL2 (CST #15071), purchased from Cell Signaling Technology, and anti-BCL2L2 (ab190952) and anti-GAPDH (ab181602) purchased from Abcam. GAPDH was used as loading control to normalize protein concentrations. Protein bands were visualized using an Odyssey Infrared Imaging system (LI‐COR Biosciences USA).

### Cell viability and apoptosis assays

Cell viability assays were performed in 96-well culture plates by seeding the cells at a density of 2 × 10^3^ cells/well and incubation at 37 °C in 5% CO_2_ overnight. At the indicated time points, 10 μl CCK8 solution (Beyotime Biotechnology) was added to each well and incubated for one hour, after which absorbance was measured at 450 nm using a plate reader. For Annexin V-FITC/PI double staining apoptosis detection, cells were collected and harvested, washed with cold PBS, adjusted to 1 × 10^6^ cells/ml in 1x binding buffer and stained with Annexin V-FITC and PI solution (BD Pharmingen, San Diego, CA, USA) for 15 min at room temperature in the dark. Finally, the stained cells were analyzed using flow cytometry (BD FACSCalibur, San Jose, CA, USA).

### TUNEL assay

Apoptotic cell death was detected using a terminal deoxyribonucleotide transferase (TdT)-mediated dUTP nick-end labeling (TUNEL) detecting system (R&D, AF835) as described previously [17].

### Transwell invasion assay

A modified double chamber (Costar, Cambridge, NY, USA) was used to test cell invasion. 1 × 10^5^ cells were seeded in the upper chamber in 100 μl serum-free DMEM, and the lower chamber was filled with 600 μl DMEM/10% FBS. After incubation for 24 h, the invaded cells were stained with crystal violet. Images were captured using a bright field microscope.

### In situ hybridization

Double digoxigenin (DIG)-labeled locked nucleic acid probes for MT1JP (TTCCTGCTGAACTCACC, RNA-Tm 84 °C), and a scrambled sequence (GTGTAACACGTCTATACGCCCA, RNA-Tm 87 °C) as a negative control (Exiqon, Vedbaek, Denmark) were used according to the manufacturer’s manual. Briefly, cells were fixed with 4% paraformaldehyde and incubated with Proteinase-K (15 μg/ml) for 10 min at 37 °C. After washing twice in phosphate buffered saline (PBS), the cells were dehydrated in ethanol, blocked in prehybridization buffer (3% bovine serum albumin [BSA]) for 30 min at 55 °C and incubated in hybridization buffer with probes (diluted at 1:2000) for 1 h at 55 °C. Next, the cells were washed with standard saline citrate buffer and blocked with 4% BSA for 1 h at room temperature. Positive signals were detected after overnight incubation with an anti-DIG primary mouse monoclonal antibody at 4 °C, followed by incubation with fluorescein isothiocyanate (FITC)-conjugated antibodies. DAPI (4′,6-diamidino-2-phenylindole) was used to stain the cell nuclei. Finally, stained cells were evaluated using laser scanning confocal microscopy.

### Luciferase reporter assay

A MT1JP fragment containing putative miR-24-3p target sites was amplified by PCR using 5’-CTCCTGCAAGAAGAGCTGC-3′ and 5’-TGCAGCAAATGGCTCAGTA-3′ primers, and cloned into the NaeI and HindIII sites of a pMIR-7 REPORT vector (Ambion). This reporter construct was named wild-type-MT1JP (WT-MT1JP). To generate a mutant reporter plasmid, we used a Generate site-directed mutagenesis system (Invitrogen) to introduce mutations into the putative miR-24-3p target sites of the wild-type-MT1JP vector. A mutant reporter (MUT-MT1JP) was constructed in which “GGCUCAGU” [71825078–718,250,786 nt] was converted into “CCGAGTCG”. The reporter plasmid was transfected into cells using Lipofectamine 2000. To correct for transfection efficiency, an empty luciferase reporter vector without the miR-24-3p target was transfected in parallel. Luciferase activities in cells were expressed as ratios of the luciferase activity of the reporter vector with the miR-24-3p targeting sequence over the one without the targeting sequence. After 48 h of transfection with or without miR-24-3p inhibitor, cells were harvested and luciferase activity was measured using a Dual-Luciferase Assay Kit (Promega, Madison, WI, USA).

### Xenograft tumor models

NOD Scid Gamma (NSG) or BALB/c nude mice (4–6 weeks old) were purchased from the Animal Core Facility of Nanjing Medical University, housed under a standard 12-h light/dark cycle and fed a standard rodent chow diet in laminar flow cabinets under specific pathogen-free conditions. All procedures regarding animal handling were in accordance with the Guide for the Care and Use of Laboratory Animals published by the National Institutes of Health (NIH). Human studies were approved by the Ethics Boards of Fuling Central Hospital of Chongqing City and written informed consent was obtained from each patient. For xenografts, approximately 5 × 10^6^ SMMC-7721 cells or transfected SMMC-7721 cells were subcutaneously implanted into the right flanks of nude mice (12 mice per group). For the establishment of patient-derived xenograft (PDX) models, fresh surgical tumor tissues (P_0_) were sectioned into 1-3 mm^3^ pieces and implanted subcutaneously into the flanks of female NSG mice. After 1 to 3 months, PDX tumors (P_1_) were transplanted (P_2_-P_n_ generations) under the skin of nude or NSG mice. When the tumors reached ~100 mm^3^ they were assigned randomly to different groups (*n* = 5/group). Xenograft tumor sizes were measured every 5 days and calculated by using the equation V (mm^3^) = (length x width^2^)/2. Mice that orally received Lenvatinib at concentrations of 0 to 100 mg/kg per day were maintained until the tumors grew to 50 mm^3^. All mice were sacrificed 30 days after the start of the Lenvatinib treatment, after which the tumor tissues were weighed and used for subsequent experiments.

### Immunohistochemistry (IHC)

For immunohistochemistry (IHC) of paraffin sections, endogenous peroxide activity was quenched through 10 min incubation in 3% H_2_O_2_, after which non-specific binding was blocked with serum. The resulting sections were incubated with an anti-Ki67 (Abcam ab245113) antibody at a 1:100 dilution overnight. Subsequently, diluted biotinylated anti-rabbit IgG (Vectastain kit) was added to the sections and incubated for 30 min. Next, Vectastain ABC reagent (Vector) and 3, 30-diaminobenzamidine (DAB) were used for color development. Counterstaining was performed using hematoxylin solution.

### Statistical analysis

The data are expressed as mean ± SEM. Statistical significance was assessed using Student’s t test. Overall survival distributions were estimated using Kaplan-Meier analysis. *P *< 0.05 was considered to be statistically significant. All experimental data were analyzed using SPSS16.0 (IBM, New York, USA) and all results were obtained from at least three separate experiments.

## Results

### Lenvatinib resistant HCC cells are refractory to Lenvatinib-induced growth inhibition and apoptosis

**Fig. 1:**
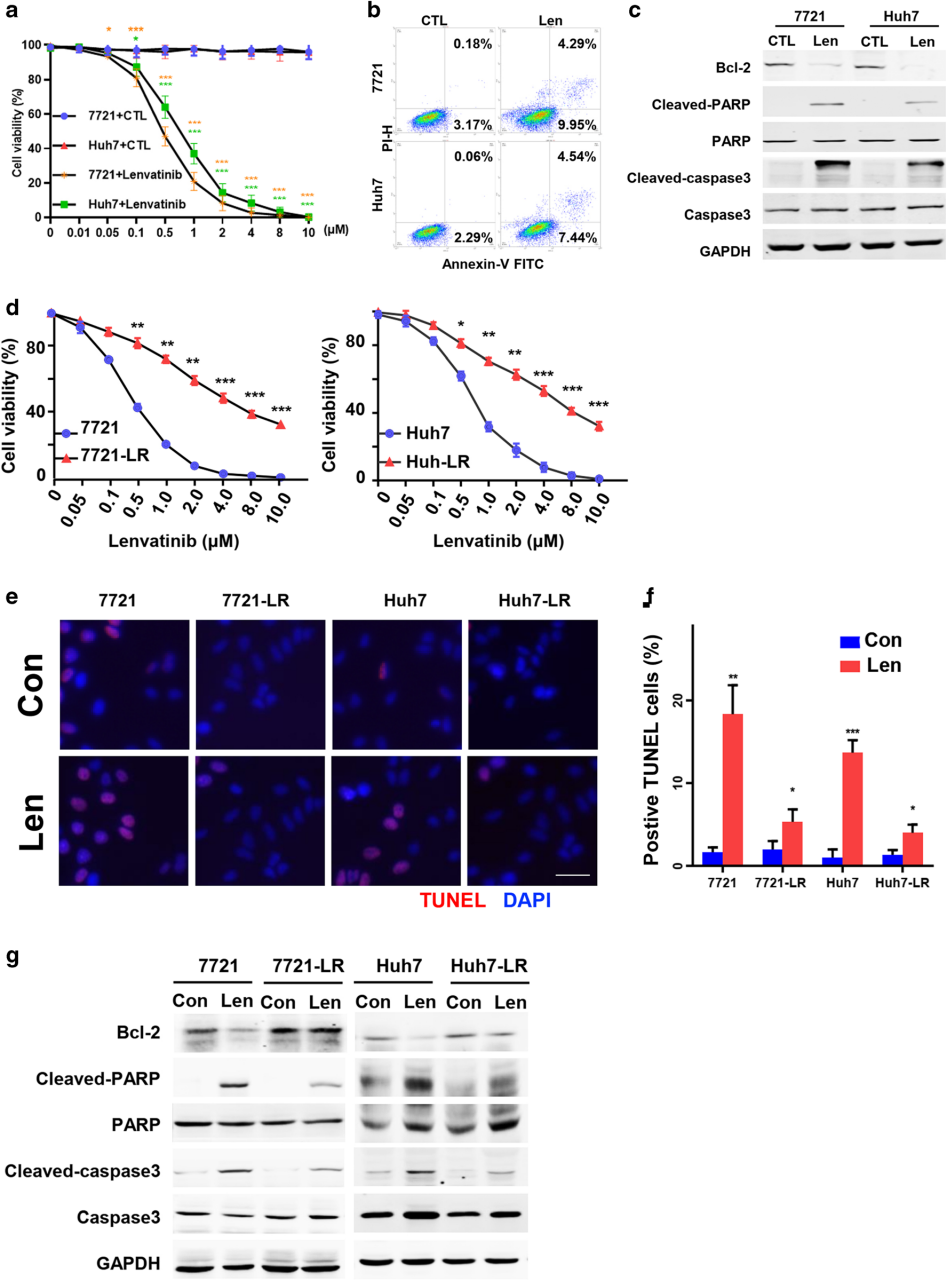
**Lenvatinib-resistant HCC cells are refractory to Lenvatinib-induced growth inhibition and apoptosis. a.** HCC cell lines SMMC-7721 and Huh7 were incubated with increasing concentrations of Lenvatinib for 48 h. Cell viability (%) was compared with corresponding untreated cells (*n* = 4). **b**-**c**. HCC cell lines were incubated with or without 0.5 μM/L Lenvatinib for 48 h. The cells were analyzed cytometrically to detect apoptosis (**b**), the rates of apoptosis were labeled, and cell lysates were immunoblotted (**c**). For Western blotting, band densities were normalized to GADPH. **d**. Lenvatinib-resistant HCC cells (7721-LR, Huh7-LR) were incubated with Lenvatinib for 48 h after which cell viabilities detected by CCK8 assay were calculated (*n* = 4). **e**-**g**. Apoptosis was detected in 7721-LR, Huh7-LR and parental 7721 and Huh7 cells, which were incubated with or without Lenvatinib (Len and Con). Results of TUNEL (**e**), its quantity (**f**) and Western blots (**g**) are presented. Scale bars: 10 μm. Compared using Student’s t test, *p < 0.05, **p < 0.01, ***p < 0.001

We found that Lenvatinib exposure inhibited the viability (Fig. 1a) and promoted the apoptosis (Fig. 1b-c) of both SMMC-7721 and Huh7 cells, and resulted in a slight variation in migration (Fig. S1A-B). The apoptotic rates of SMMC-7721 and Huh7 cells were 4.2- and 5.1-fold increased (14.24% vs 3.35% and 11.98% vs 2.35%) after exposure to 0.5 μM/L Lenvatinib, respectively (Fig. 1b). These results were supported by the expression of two key apoptotic proteins, caspase-3 and PARP and an anti-apoptotic protein, Bcl-2, i.e., Lenvatinib downregulated Bcl-2 and promoted the cleavage of caspase-3 and PARP (Fig. 1c).

Two Lenvatinib-resistant HCC cells lines, 7721-LR and Huh7-LR, were established by first determining the Lenvatinib IC_50_ values of SMMC-7721 and Huh7 cells (which were 0.42 and 0.60 μM, respectively; Fig. 1a), and next incubation of the cells with gradually increasing concentrations of Lenvatinib for 6 to 7 months. The Lenvatinib-resistant properties of the LR-HCC cells were confirmed by comparing their viabilities with the parental HCC cells following incubation with various concentrations of Lenvatinib (Fig. 1d). After acquiring resistance, 7721-LR and Huh7-LR cells exhibited increased anti-apoptosis and higher migration capacities (Fig. 1e-g and S1C-D).

Even without Lenvatinib stimulation, the apoptotic rates of SMMC-7721 and Huh7 cells were 2.1- and 2.4-fold higher (5.25% vs 2.53% and 4.01% vs 1.70%) than those of 7721-LR and Huh7-LR cells (Fig. S1E). After exposure to 0.5 μM Lenvatinib, the apoptotic rates of 7721-LR and Huh7-LR cells decreased by 1.9- and 2.0-fold (7.66% vs 14.31% and 6.06% vs 12.29%) compared to those of SMMC-7721 and Huh7 cells (Fig. S1E). These results were confirmed using a TUNEL assay (Fig. 1e-f). In addition, we found that acquiring resistance to Lenvatinib led to inhibition of the apoptosis pathway and its downstream factors including caspase-3 and PARP (Fig. 1g).

### Lenvatinib-resistant HCC cells overexpress Lnc MT1JP

**Fig. 2:**
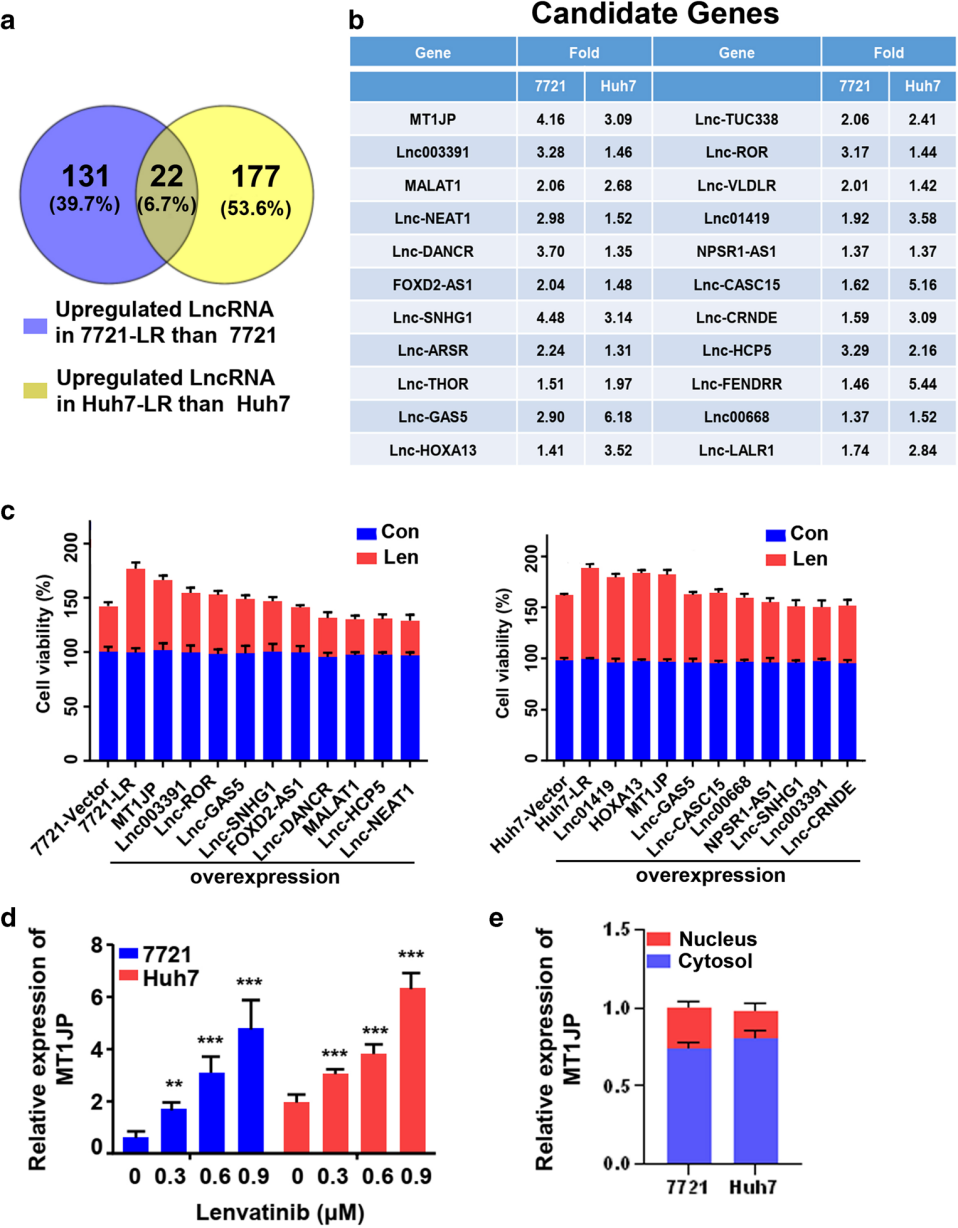
**LINC-MT1JP is upregulated in Lenvatinib-resistant HCC cells. a**-**b**. Venn diagram showing overlap of differentially upregulated expression of genes in LR-7721 cells relative to control versus LR-Huh7 cells relative to control. Details of the identified candidate genes are listed in (**b**). **c**. SMMC-7721 or Huh7 cells were subjected to overexpression of these 10 candidate genes after which cell viabilities (%) were determined and compared with LR-HCC cells treated with Lenvatinib (*n* = 3). **d**. qRT-PCR confirmation of Lnc-MT1JP (MT1JP) expression with increasing concentrations of Lenvatinib for 48 h (*n* = 4). **e**. Total RNA was extracted from nuclear and cytoplasmic fractions after which the expression of MT1JP was determined by qRT-PCR and normalized. U1 was used as internal nuclear control. GAPDH was used as a cytoplasmic control. Compared using Student’s t test, *p < 0.05, **p < 0.01, ***p < 0.001

RNA sequencing (RNA-seq) was used to asses transcriptional changes induced by Lenvatinib resistance in the respective HCC cell lines (Fig. S2A). Subsequent GO analysis revealed that 22 (6.7%) up-regulated long non-coding RNAs (Lnc-RNAs) differentially expressed in 7721-LR cells relative to parental SMMC-7721 cells were also differentially expressed in Huh7-LR cells relative to parental Huh7 cells (Fig. 2a-b). Expression alterations of the top 10 of these 22 genes in the two Lenvatinib-resistant HCC cell lines were confirmed by qRT-PCR (Fig. S2B-C). To reveal the functions of these Lnc-RNAs, the top Lnc-RNAs were exogenously overexpressed in both SMMC-7721 and Huh7 cells after which their viability after exposure to Lenvatinib was assessed. The results obtained significantly pointed to a critical role of Lnc-MT1JP (MT1JP) on HCC cell viability (Fig. 2c). In addition we found that, after incubation for 48 h, the expression of MT1JP increased with increasing concentrations of Lenvatinib both in SMMC-7721 and Huh7 cells (Fig. 2d). We next examined the intracellular location of MT1JP by assessing its expression in nuclear and cytoplasmic fractions of SMMC-7721 and Huh7 cells using in situ hybridization with MT1JP-specific probes. The results indicated that 75%–80% of MT1JP was located in the cytoplasmic fractions of the HCC cells (Fig. 2e and S2D).

### MT1JP contributes to Lenvatinib resistance in HCC cells by inactivating the apoptosis pathway

**Fig. 3:**
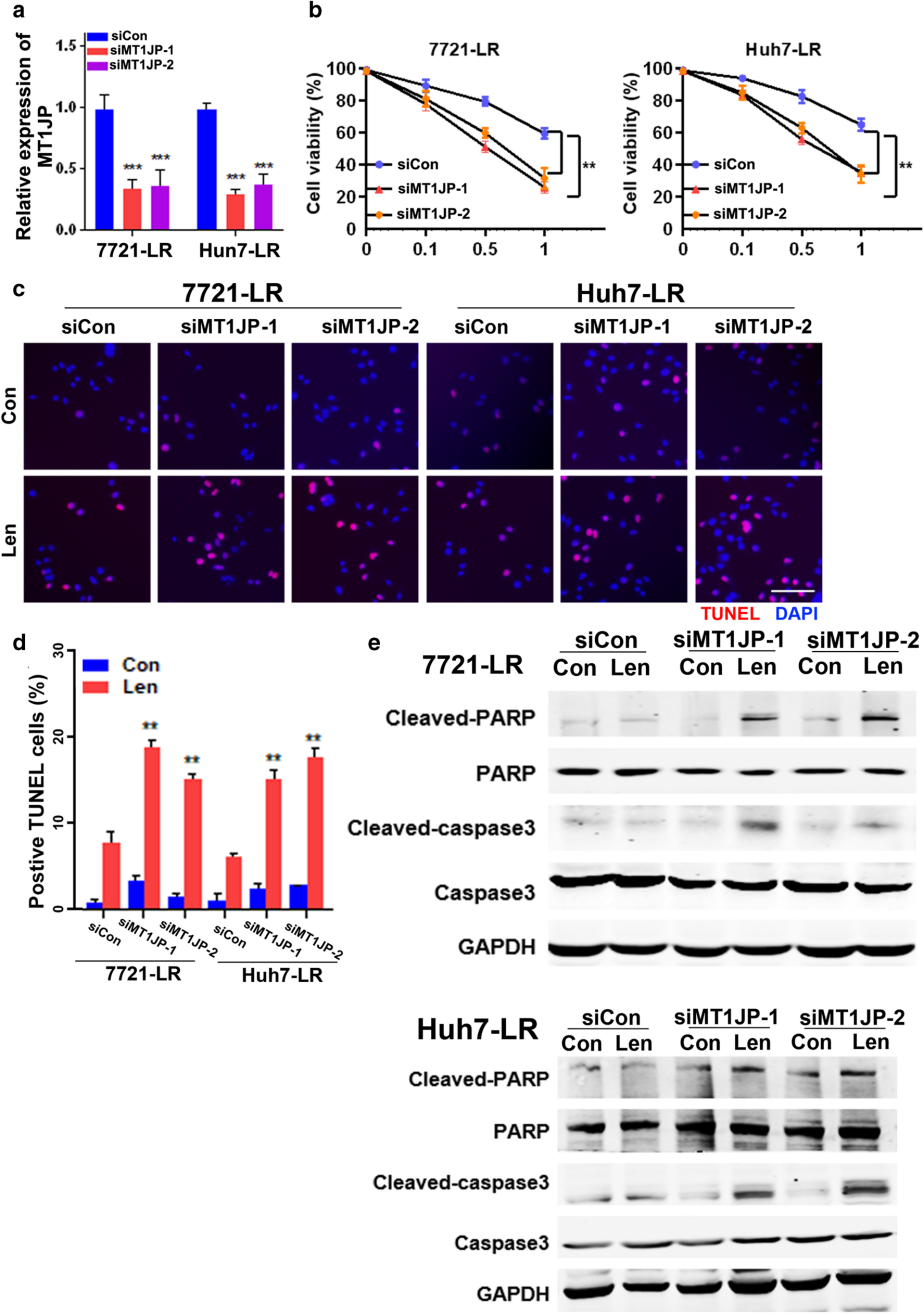
**MT1JP confers poor Lenvatinib response on HCC cells through inhibiting apoptosis. a.** LR-7721, LR-Huh7 and parental HCC cells were transfected with either control vector (siCon) or two siRNAs targeting MT1JP (siMT1JP-1 and siMT1JP-2) and incubated for 48 h in the absence or presence of Lenvatinib (0.5 μM) (Con and Len). The transfection efficiencies were confirmed by qRT-PCR. **b.** Viabilities (%) of the above cells (**a**) treated with or without Lenvatinib (Len and Con) were calculated (*n* = 3). **c**. Representative images taken from the above cells (**a**) incubated with or without Lenvatinib and stained by TUNEL (red) and DAPI (blue) (Scale bars: 10 μm). **d**. Quantification of TUNEL positive cells in (**c**). **e**. Lysates from the above cells (**a**) incubated with or without Lenvatinib were subjected to Western blot analysis. Band densities were normalized to GADPH. Compared using Student’s t test, *p < 0.05, **p < 0.01, ***p < 0.001

We next examined the relationship between MT1JP expression and Lenvatinib resistance. To this end, mock (siCon) and anti-MT1JP (siMT1JP) siRNA transfected LR-HCC cells were incubated with Lenvatinib for 48 h. The efficiency of siRNA-mediated silencing was confirmed by qRT-PCR (Fig. 3a). Next, we found that in LR-HCC cells, MT1JP silencing resulted in alterations in cell viability (Fig. 3b), leading to a 2.2-fold upregulation of the apoptotic rate (Fig. 3c-d) and an induction of the apoptosis signaling pathway, after Lenvatinib exposure (Fig. 3e). The anti-apoptotic abilities of LR-HCC cells were found to be decreased after MT1JP silencing, which was confirmed by expression analysis of key apoptotic proteins (Fig. 3e). Both MT1JP overexpressing SMMC-7721 and Huh7 cells acquired resistance to Lenvatinib (Fig. S3A-B). Concordantly, we found that exogenous MT1JP overexpression (MT1JP) decreased the apoptotic rates by 2.1- and 2.4-fold compared to the mock group (vector) (Fig. S3C). MT1JP overexpression also led to downregulation of the expression of cleaved-Caspase-3 and PARP (Fig. S3D). Interestingly, overexpression of MT1JP did not significantly change HCC cell migration after Lenvatinib treatment (Fig. S3E).

### MT1JP serves as a ceRNA for miR-24-3p to regulate HCC cell apoptosis

**Fig. 4:**
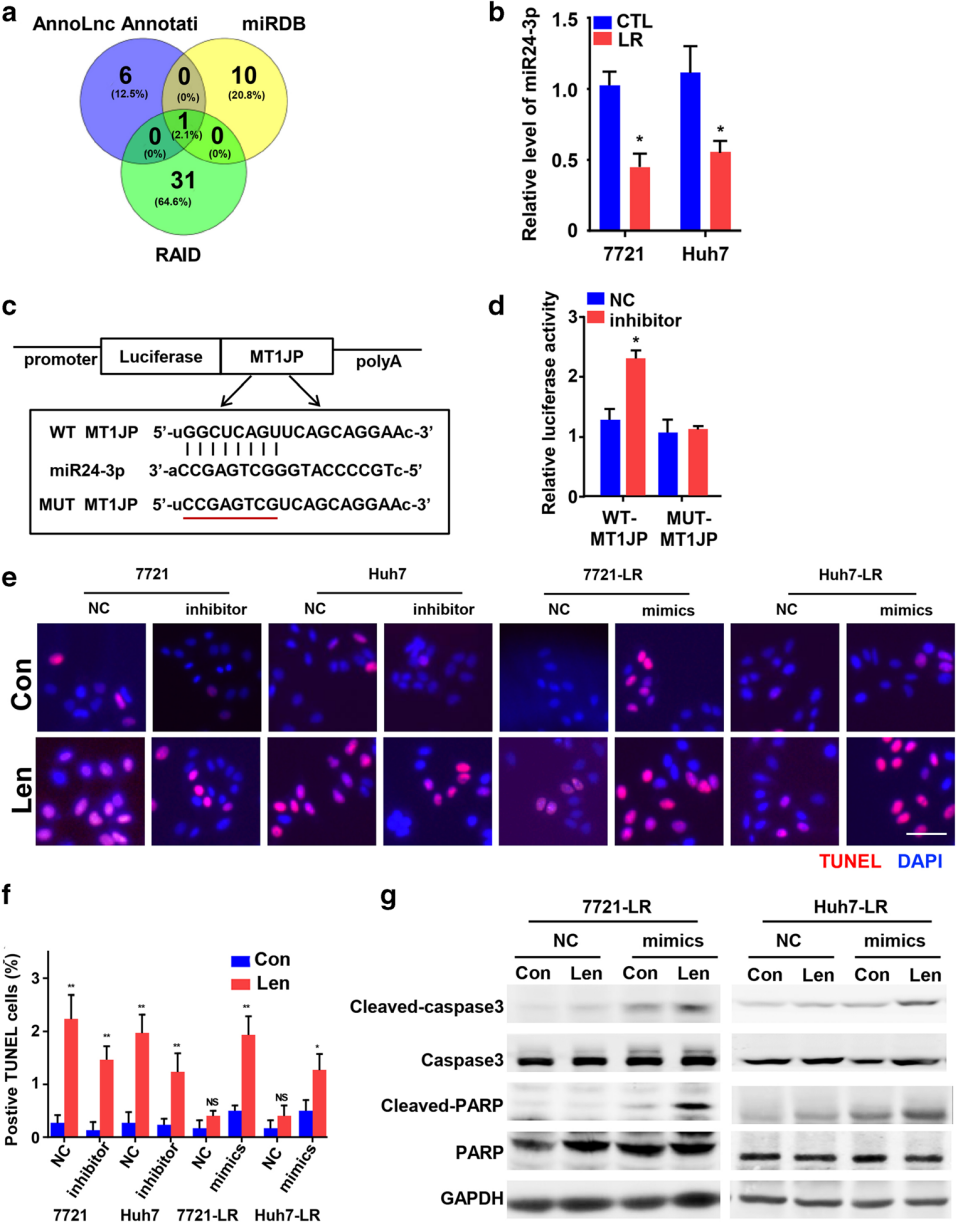
**MT1JP acts as a ceRNA for miR-24-3p to regulate the apoptotic signaling pathway. a**. Venn diagram showing overlapping miRNAs that are predicted to bind to MT1JP. **b**. Expression analysis of miR-24-3p measured in Lenvatinib resistant and parental HCC cells by qRT-PCR. **c**. Binding sequences of MT1JP and miR-24-3p based on bioinformatics analysis and binding sites of WT (WT-MT1JP) and mutant (Mut-MT1JP) regions. **d**. Dual-luciferase reporter assays to examine the potential interaction of MT1JP and miR-24-3 (*n* = 4). **e**-**g**. SMMC-7721 and Huh7 cells were transfected with miR-NC (NC) or miR-24-3p inhibitor (inhibitor), while LR-7721 and LR-Huh7 cells were transfected with miR-NC (NC) or miR-24-3p mimics (mimics). Apoptosis through TUNEL (**e**-**f**) and Western blotting (**g**) assays was detected in these transfected cell lines. (Scale bars: 10 μm). Data represent three independent experiments. Compared using Student’s t test, *p < 0.05, **p < 0.01, ***p < 0.001

Previously, it has been reported that LncRNAs can regulate expression levels and biological functions of specific miRNAs, either acting as molecular sponges or as competing endogenous RNAs (ceRNAs) [18]. In order to identify possible targets of MT1JP, we used miRDB (http://mirdb.org/miRDB/custom.html), AnnoLnc (http://annolnc.cbi.pku.edu.cn) and RAID (http://www.rna-society.org/raid/) online prediction tools to find candidate miRNAs that may be regulated by MT1JP (Fig. 4a). Of the miRNAs that fit the criteria, only miR-24-3p emerged as a candidate, as its predicted binding sites were shared by MT1JP (Fig. S4A). Next, we assessed the expression of miR-24-3p in control and LR cells and found that its expression was lower in cell lines in which the MT1JP mRNA levels were relatively higher (Fig. 4b). For a further confirmation of a putative interaction between MT1JP and miR-24-3p, we subcloned MT1JP (wild type [WT] and mutant [Mut]) downstream of the firefly luciferase gene into a pmirGLO vector, based on online RAID analysis (Fig. 4c), and next performed a luciferase reporter assay. We found that anti-miR-24-3p markedly increased the luciferase activity of the WT-MT1JP construct, but not of the Mut-MT1JP construct (Fig. 4d). These results indicate that miR-24-3p can bind directly to MT1JP at its miRNA recognition site.

We next explored the effect of miR-24-3p expression on apoptosis and Lenvatinib sensitivity of HCC cells. To this end, we transfected miR-NC/miR-24-3p inhibitor into SMMC-7721 or Huh-7 cells and miR-NC/miR-24-3p mimics into LR-HCC cells. The respective expression effects were confirmed by qRT-PCR (Fig. S4B). Since miR-24-3 inhibition did not affect the expression of MT1JP (Fig. S4C), we first analyzed the relation between cell viability in response to Lenvatinib exposure and relative miR-24-3p expression in the HCC cells and found that miR-24-3p overexpression decreased the viability of Lenvatinib treated LR-HCC cells (Fig. S4D). Opposite effects were observed in HCC cells after inhibition of miR-24-3p (Fig. S4E). Subsequently, we investigated the apoptotic rates after transfection. Using TUNEL and Western blotting assays, we found that miR-24-3p had a negative effect on the apoptosis pathway (Fig. 4e-g and Fig. S4F). Taken together, these data indicate that MT1JP serves as a ceRNA for miR-24-3p to regulate the apoptosis pathway in HCC cells.

### miR-24-3p regulates HCC cell apoptosis suppression through BCL2L2

**Fig. 5:**
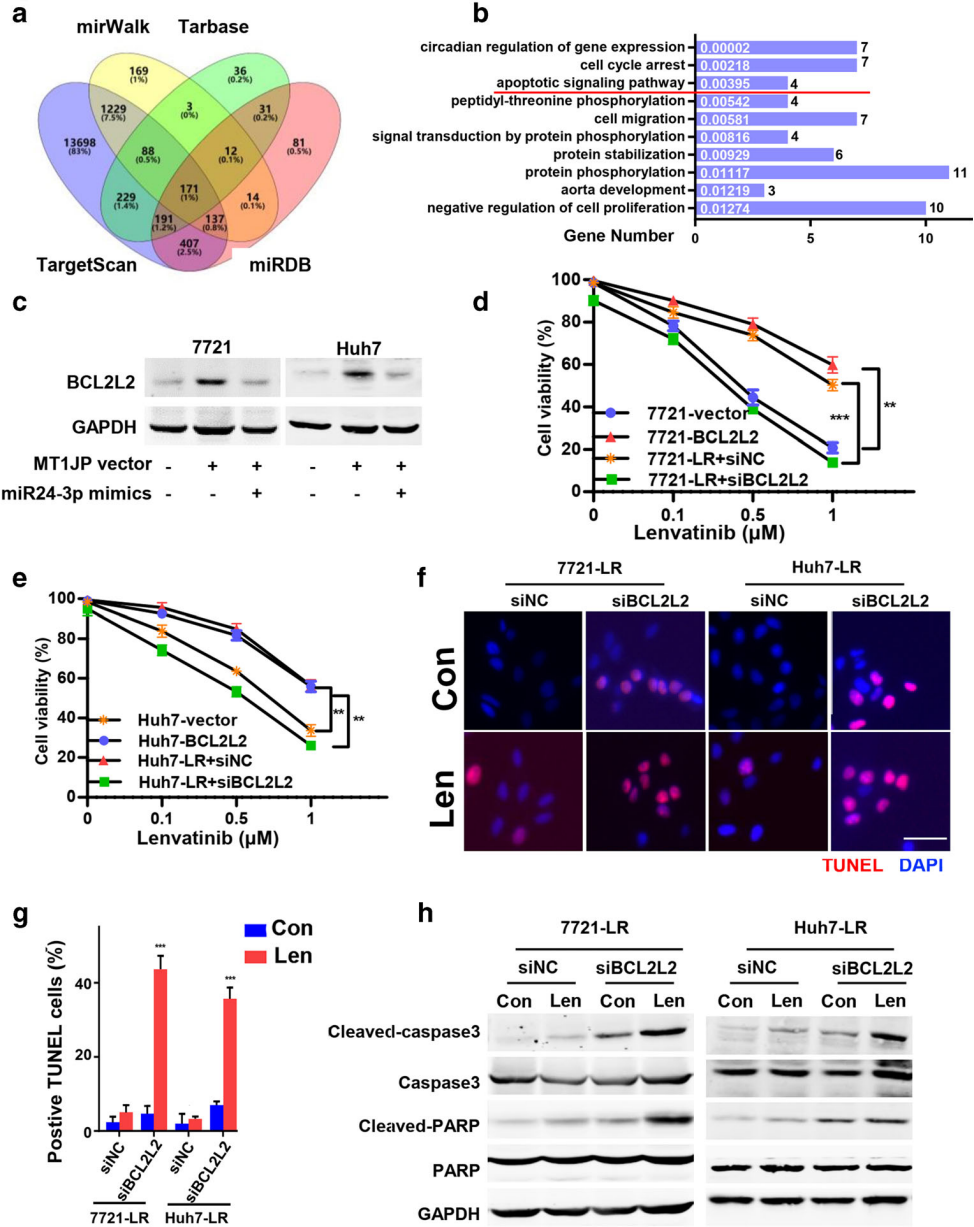
**BCL2L2 suppression, mediated by miR-24-3p, is involved in MT1JP-mediated Lenvatinib tolerance. a**. Venn diagram showing overlapping genes that are predicted to bind to miR-24-3p. **b**. GO enrichment graph of the common 171 genes predicted in (**a**). *P* values (white) and different gene numbers (black) are listed in the graph. **c**. HCC cell lines were transfected with either MT1JP vector or miR-24-3p mimics. Expression of BCL2L2 was detected by Western blotting. **d**-**e**. SMMC-7721 and Huh7 cells were transfected with control vector (vector) or BCL2L2, while LR-7721 and LR-Huh7 cells were transfected with control siRNA (siNC) or BCL2L2 siRNA (siBCL2L2). The viability (%) of these transfected cells incubated with Lenvatinib (5 μM) was calculated. **f**-**h**. Apoptosis through TUNEL (**f**-**g**) and Western blotting (**h**) assays was detected in the above cells (**e**). Data represent three independent experiments. (Scale bars: 10 μm). Compared using Student’s t test, *p < 0.05, **p < 0.01, ***p < 0.001

To assess the role of miR-24-3p in the apoptosis regulatory loop, online prediction tools were used to identify candidate targets. We identified 171 genes as candidates (Fig. 5a). Further GO analysis revealed that 4 of the 171 genes were associated with the apoptosis pathway (Fig. 5b). The expression of all four genes tended to be increased in miR-24-3p silenced HCC cells (Fig. S5A), but only cells overexpressing Bcl-2 like 2 (BCL2L2) exhibited a better cell viability after exposure to Lenvatinib compared to the other three genes (Fig. S5B-C). Moreover, the expression of BCL2L2 was dramatically increased after MT1JP overexpression, which could be reversed after miR-24-3p mimics transfection (Fig. 5c). Conversely, we found that overexpression of miR-24-3p in LR-HCC cells led to a decreased expression of BCL2L2, which could be reversed by transfection of a wild type MT1JP vector but not of a mutant MT1JP vector (Fig. S5D), which is in accordance with previous results. To better understand the role of BCL2L2 in LR formation, BCL2L2 was overexpressed in SMMC-7721 and Huh7 cells and silenced in LR-7721 and Huh7 (siBCL2L2) cells. The respective transfection efficiencies were confirmed (Fig. S5E). We first analyzed the correlation between IC_50_ in response to Lenvatinib and BCL2L2 expression in the transfected LR-HCC cells and found that these cells became sensitive to Lenvatinib treatment after inhibition of BCL2L2 expression (Fig. 5d-e). Compared to the control group, BCL2L2 silencing increased the sensitivity of HCC cells to Lenvatinib via apoptosis induction, whereas exogenous overexpression of BCL2L2 in LR-HCC cells decreased the levels of cleaved-Caspase 3 and PARP (Fig. 5f-h). Concordant results were obtained with HCC cells overexpressing BCL2L2 (Fig. 5d-e and S5F-H). These data suggest that miR-24-3p-mediated BCL2L2 suppression is involved in MT1JP-induced poor Lenvatinib sensitivity.

### The MT1JP/miR-24–3p axis contributes to in vivo Lenvatinib resistance in HCC PDX models

**Fig. 6:**
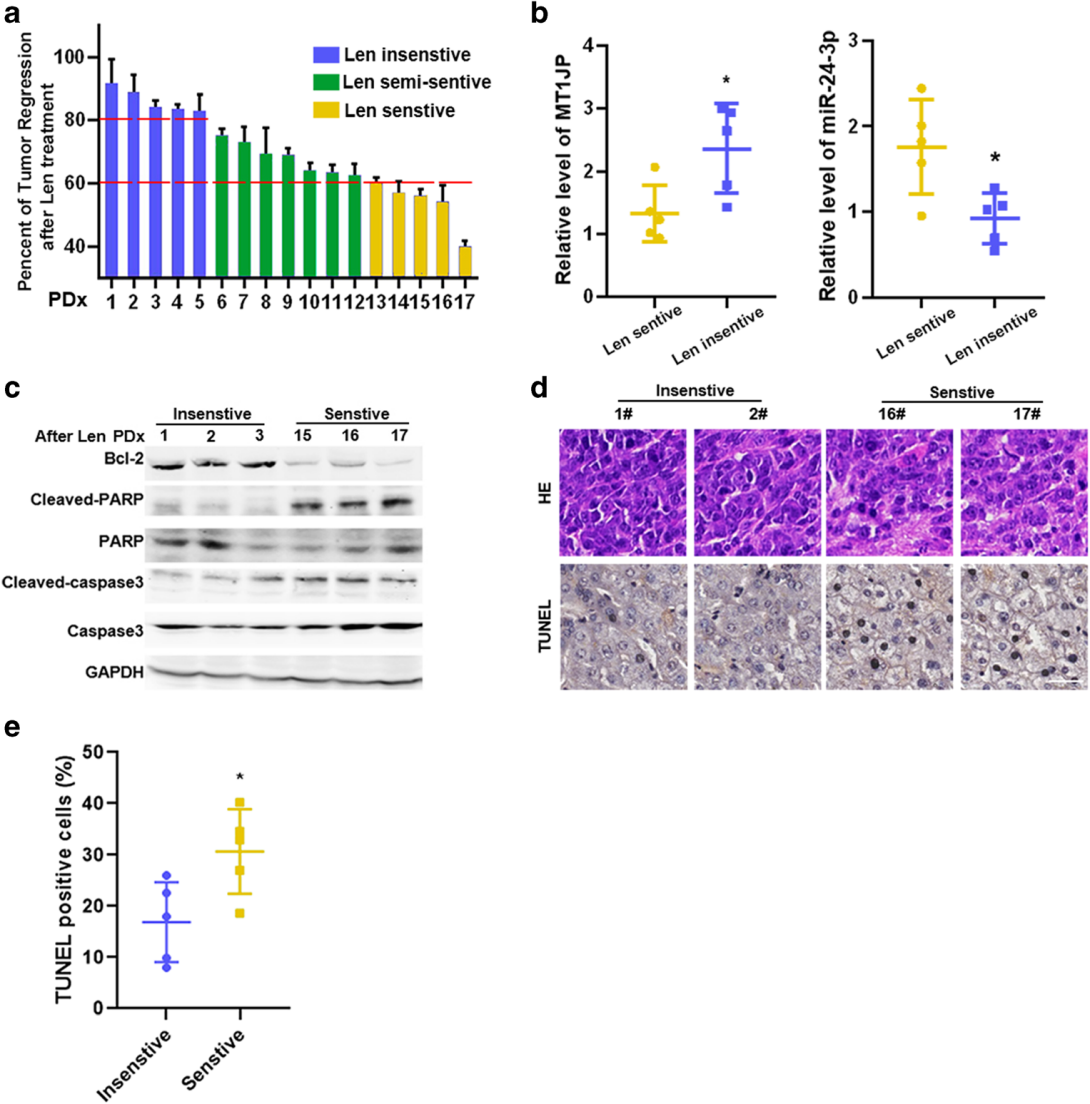
**MT1JP/miR-24–3p axis contributes to Lenvatinib resistance in a HCC PDX model. a.** Seventeen fragments of PDX tumors from HCC patients were used for Lenvatinib sensitivity screening. NSG mice received oral Lenvatinib administration (50 mg/kg) once the founder PDX tumors reached ~ 1.0 cm in diameter. The percentage of tumor regression was calculated as reduced volume after Lenvatinib treatment, normalized to the primary tumor volume. A > 40% decrease in tumor volume was termed as Lenvatinib sensitive (Len sensitive, 5/17, 29.4%), and a < 20% decrease as Lenvatinib insensitive (Len insensitive, 5/17, 29.4%). **b**. Relative expression levels of MT1JP and miR-24-3p in Len sensitive and insensitive groups measured by qRT-PCR. **c**. Lysates from Len sensitive and insensitive PDX cases after Lenvatinib treatment were subjected to Western blot analysis. Band densities were normalized to GADPH. **d**-**e**. Representative images of H&E staining and TUNEL staining are shown. (Scale bars: 50 μm) Quantification of TUNEL positive cells in (**c**) is shown in (**e**). Compared using Student’s t test, *p < 0.05, **p < 0.01, ***p < 0.001

We processed 26 fresh HCC specimens from different patients and successfully established 17 PDX models. The xenografted NSG mice orally received Lenvatinib (50 mg/kg) once the PDX tumors reached about 1.0 cm in diameter. The percentage of tumor regression was calculated after continuous administration of Lenvatinib for two weeks. A > 40% decrease in tumor volume was designated as Lenvatinib sensitive (5/17, 29.4%), and a < 20% decrease as Lenvatinib insensitive (5/17, 29.4%, Fig. 6a and S6 A). The Lenvatinib sensitive group exhibited a lower expression of MT1JP and a higher expression of miR-24-3p compared to the Lenvatinib insensitive group (Fig. 6b), which is in accordance with the in vitro HCC cell line data. Moreover, Western blotting and IHC evaluation of the apoptosis pathway indicated that in the Lenvatinib sensitive group the apoptosis signals were induced to promote tumor degradation (Fig. 6c-e). These results further confirm our in vitro findings in HCC cell lines.

### MT1JP and BCL2L2 overexpression attenuate in vivo HCC sensitivity to Lenvatinib

**Fig. 7:**
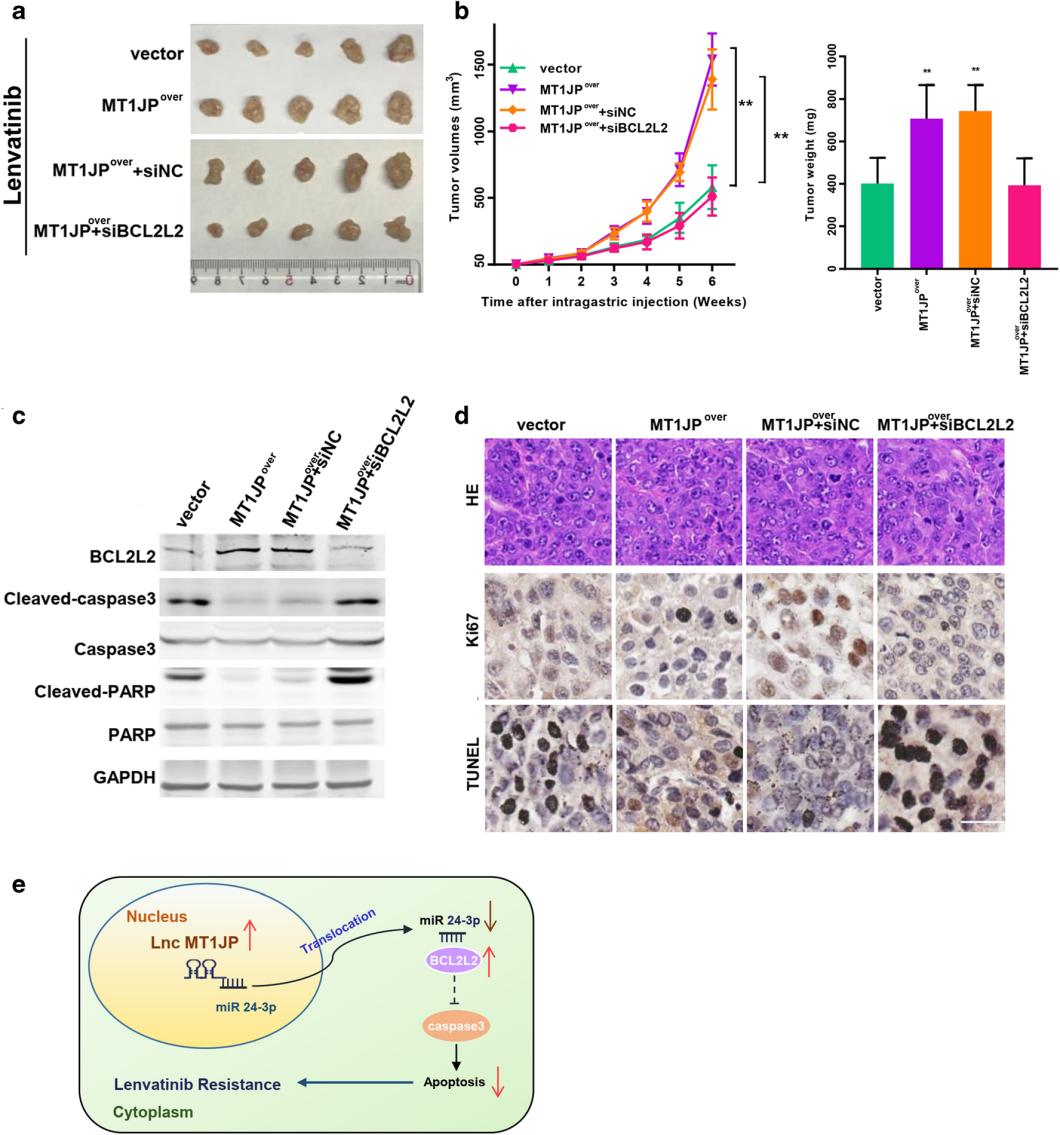
**MT1JP or BCL2L2 overexpression attenuate sensitivity to Lenvatinib in vivo. a.** MT1JP (MTJ1P^over^) or control vector (vector) were overexpressed in SMMC-7721 cells followed by interfering with BCL2L2 (MT1JP^over^ + siBCL2L2) or its control (MT1JP^over^ + siNC). These cells were implanted into nude mice. When the average tumor volume reached ~ 50 mm^3^, the mice were randomly subdivided into four groups and administered 50 mg/kg Lenvatinib daily. Tumor sizes were calculated every 5 days. Images of subcutaneous xenograft tumors were taken. **b**. Growth curves (left) or tumor burden (right) of the subcutaneous xenograft tumors were calculated (*n* = 12 mice for each group). **c**. Lysates from the above tumors (**a**) were subjected to Western blot analysis. **d**. Representative images of H&E staining, immunohistochemical staining (for Ki67) and TUNEL staining are shown. (Scale bars: 50 μm). **e**. Schematic overview of MT1JP and apoptosis regulatory signaling

To verify the effects of MT1JP and BCL2L2 on sensitivity to Lenvatinib in vivo, we employed a xenograft model with SMMC-7721 cells. After the volume of subcutaneous tumors reached ~50 mm^3^, different doses of Lenvatinib were orally administered after which the tumor volumes were assessed every 5 days. Considering the size of the tumors, a dose of 50 mg/kg was found to be appropriate for the mice (Fig. S7A-F). In addition, SMMC-7721 cells with stable MT1JP overexpression or BCL2L2 inhibition and SMMC-7721 cells transfected with an empty vector or inhibition control as negative controls were injected into mice, which were not treated with 50 mg/kg Lenvatinib until the tumors reached ~50 mm^3^. Consistent with our in vitro observations, we found that tumors overexpressing MT1JP or with BCL2L2 inhibition grew faster than those derived from control cells (Fig. 7a-b). We also found no significant alternations after synchronous MT1JP overexpression and BCL2L2 inhibition compared to the control group (Fig. 7a-b). These results further suggest that MT1JP can promote Lenvatinib resistance via the miR-24-3p/ BCL2L2 axis. In addition, we applied Western blotting to assess apoptosis pathway alterations in the tumors. We found that tumors derived from stably MT1JP- or BCL2L2-transfected SMMC-7721 cells presented with a significantly reduced apoptosis compared with tumors derived from control cells, and that this effect was reversed by synchronous MT1JP overexpression and BCL2L2 inhibition (Fig. 7c). Using IHC, similar results were obtained (Fig. 7d). Therefore, we conclude that our in vivo experiments underscore the results of the in vitro experiments.

## Discussion

Lenvatinib is a novel TKI drug for advanced HCC and is, next to Sorafenib, approved by many international and national authorities. The survival benefit from Lenvatinib may, however, be hampered by therapy resistance as is true for almost all TKIs [19]. Therefore, it is essential to explore the mechanisms underlying Lenvatinib resistance in HCC. In the present study, we found that Lenvatinib-induced upregulation of Lnc-RNA MT1JP (MT1JP) contributes to Lenvatinib resistance (LR) by inhibiting activation of the apoptosis pathway via the miR-24-3p/BCL2L2 axis. MT1JP silencing decreased the effect of Lenvatinib against LR-HCC cells that were refractory to Lenvatinib-induced proliferation inhibition and apoptosis in vitro and in vivo. We showed that Lenvatinib promoted the apoptosis pathway in a concentration-dependent manner and that LR increased the anti-apoptotic effects of Lenvatinib in both SMMC-7721 and Huh7 cells, which implies that LR formation is probably associated with inhibition of the apoptosis pathway. Several other studies have shown that the apoptosis pathway may be activated after Lenvatinib stimulation [20–22]. Although some studies reported that Lenvatinib did not dose-dependently suppress tumor growth and apoptosis in cell lines [14], it should be noted that instant culturing with Lenvatinib may not be enough to affect the apoptosis pathway [20]. We found that Lenvatinib-treated cells showed a marked suppression of viability and upregulation of apoptosis signaling, similar to previous studies [20, 21]. This effect could be reversed when the HCC cells acquired resistance to Lenvatinib.

The proposed mechanisms by which MT1JP activates the anti-apoptosis pathway and its regulation by miR-24-3p in LR-HCC cells are depicted schematically in Fig. 7e. MT1JP has previously been reported to act as a tumor suppressor in breast and bladder cancer [23, 24] and it has been reported that MT1JP may inhibit the proliferation, invasion and migration of tumor cells [25, 26]. Zhu et al. reported that MT1JP could inhibit the tumorigenesis and enhance the cisplatin sensitivity of breast cancer cells through competitively binding to miR-24-3p [22]. In this study, overexpression of MT1JP in breast cancer cells significantly inhibited their proliferation and enhanced their cisplatin sensitivity, which seems to be discordant with our results. It should be noted, however, that breast cancer cells are sensitive to cisplatin when proliferating, and that blocking the cell cycle will affect the efficacy of cisplatin. In contrast, HCC cells are quite insensitive to chemotherapy and Lenvatinib inhibits HCC proliferation through the apoptosis signaling pathway, which may explain the differences in results between breast cancer and HCC cells.

It is well-known that cross-talk between Lnc-RNAs and miRNAs is involved in many biological processes [27]. Therefore, in the present study, we further explored the interaction between MT1JP and miR-24-3p in the apoptosis pathway. Previously, miR-24-3p has been shown to play an important role in regulating cell growth and metastasis in various types of cancer [22, 28, 29]. MiR-24-3p has also been identified as a key miRNA associated with activation of the apoptosis pathway [29]. Intriguingly, our study revealed a novel regulatory pathway, in which Lenvatinib induces expression of MT1JP, acting as a competing endogenous RNA with miR-24-3p, regulating the anti-apoptosis protein BCL2L2. BCL2L2 is a member of the Bcl-2 protein family, which act as anti-apoptotic regulators and reduce apoptosis under cytotoxic conditions [30–32]. Our results indicate that high expression of BCL2L2 leads to resistance to Lenvatinib and that downregulation of this protein may increase HCC sensitivity to Lenvatinib in vivo and in vitro*,* which agrees with a previous study showing that Bcl-2 can effectively curb ovarian cancer cell apoptosis through resistance to cisplatin [33]. This notion may point at a promising therapeutic target for the treatment of Lenvatinib-resistant HCC.

One caveat of the present study is that it does not address the mechanism of invasion of LR HCC cells and the possible role of the MT1JP/miR-24-3p/BCL2L2 axis therein. In addition, the results obtained in the cell and animal models have not been validated in primary tumor tissues collected from HCC patients who have developed Lenvatinib resistance. Thus, validation in clinical LR-HCC tissues needs to be performed in future studies.

## Conclusions

In the present study we show that Lnc-RNA MT1JP contributes to HCC Lenvatinib resistance by inhibiting apoptosis through regulating the miR-24-3p/BCL2L2 axis. MT1JP silencing enhanced the efficacy of Lenvatinib in suppressing LR-HCC cell propagation by promoting apoptosis in vitro in cell models and in vivo in animal models. MiR-24-3p was able to suppress the expression of BCL2L2, which is an anti-apoptotic regulator, and inhibited HCC cell resistance to Lenvatinib treatment. Our results suggest that MT1JP or BCL2L2 may serve as targets for overcoming Lenvatinib resistance in HCC.

## Supplementary Information

ESM 1 (DOCX 1487 kb)

## Data Availability

The data used and analyzed during the current study are available from the corresponding author upon reasonable request.

## Abbreviations

BCL2L2: Bcl-2 Like 2
ceRNA: competing endogenous RNA
FGF: fibroblast growth factor
GAPDH: glyceraldehyde-3-phosphate dehydrogenase
HBV: hepatitis B virus
HCV: hepatitis C virus
HCC: hepatocellular carcinoma
LR: Lenvatinib resistance
MT1JP: Lnc-MT1JP
PDGF: platelet derived growth factor
PARP: poly ADP-ribose polymerase
RNA-seq: RNA sequencing
RNA-seq: RNA sequencing
RT-PCR: real time-polymerase chain reaction
TKI: tyrosine kinase inhibitor
TUNEL: TdT-mediated dUTP Nick-End Labeling
WB: western blot
VEGF: vascular endothelial growth factor.
