# Supplementary material for: MT1JP-mediated miR-24-3p/BCL2L2 axis promotes Lenvatinib resistance in hepatocellular carcinoma cells by inhibiting apoptosis
Source: Cell Oncol (Dordr). 2021 May 11;44(4):821–34. doi: 10.1007/s13402-021-00605-0 (PMC8338827; doi:10.1007/s13402-021-00605-0)
Supplement: Supplementary file 1 — (DOCX 1487 kb) [file 13402_2021_605_MOESM1_ESM.docx]

**Supplementary Table 1. Sequences of the primers**

| **ID** | **Gene** | **Primersequence** |
| --- | --- | --- |
| **1** | **MT1JP** | **F:5'-CTCCTGCAAGAAGAGCTGC-3'** |
| **2** | **KIF9-AS1** | **R:5'-TGCAGCAAATGGCTCAGTA-3'**  **F:5'-GCACCTTCCTTTCTTGCCAA-3'**  **R:5'-TCTTCCTCCACATCTGCCTG-3'** |
| **3** | **MALAT1** | **F:5'-AAAGCAAGGTCTCCCCACAA-3'**  **R:5'-GGTCTGTGCTAGATCAAAAGGCA-3'** |
| **4** | **Lnc-NEAT1** | **F:5'-TGTCCCTCGGCTATGTCAGA-3'**  **R:5'-GAGGGGACGTGTTTCCTGAG-3'** |
| **5** | **Lnc-DANCR** | **F:5'-GCGCCACTATGTAGCGGGTT-3'**  **R:5'-TCAATGGCTTGTGCCTGTAGTT-3'** |
| **6** | **FOXD2-AS1** | **F:5'-CTCACATCCGGCGGCT-3'**  **R:5'-GCTGTTCATGATATGTGC-3'** |
| **7** | **Lnc-SNHG1** | **F:5'-TAACCTGCTTGGCTCAAAGGG-3'**  **R:5'-CAGCCTGGAGTGAACACAGA-3'** |
| **8** | **Lnc-MIAT** | **F:5'-GAGGGAAGTTCTGAGCTTGG-3'**  **R:5'-CCTTTCTTCTGGGCTGAGAC-3'** |
| **9** | **Lnc-THOR** | **F:5'-CAAGGTGCTTCTCTCTGGATTT-3'**  **R:5'-GCCAAAGTCATTTGTTGGGTAT-3'** |
| **10** | **Lnc-GAS5** | **F:5'-TGGAGGATCGGATTTTAGCAAACT-3'**  **R:5'-CTATGGGATCGGGCAGTGCAAAGT-3'** |
| **11** | **Lnc-HOXA13** | **F:5'-GAACGGCCAAATGTACTGCC-3'**  **R:5'-GTATAAGGCACGCGCTTCTTTC-3'** |
| **12** | **Lnc-TUC339** | **F:5'-GATGAGGCCCCGAGTTTAAT-3'**  **R:5'-AGATGGAGGATCGGTGTGAA-3'** |
| **13** | **Lnc-ROR** | **F:5'-GGAGAGGAAGCCUGAGAGU-3'**  **R:5'-ACUCUCAGGCUUCCUCUCC-3'** |
| **14** | **Lnc-VLDLR** | **F:5'-AGCAGTCACATTCATCGCAC-3'**  **R:5'-GAGGAATAGGTGCGAACTGC-3'** |
| **15** | **Lnc-HULC** | **F:5'-AACCTCCAGAACTGTGAT-3'**  **R:5'-CATAATTCAGGGAGAAAG-3'** |
| **16** | **Lnc-FAL1** | **F:5'-CCTGGCCAAGAAGCTCATAC-3'**  **R:5'-TGAGGACACCGACTACTGAGAA-3'** |
| **17** | **Lnc-CASC15** | **F:5'-CACACGCATGGAAAACCCAG-3'**  **R:5'-GAGGACCTGAGCTGTAAGCC-3'** |
| **18** | **Lnc-CRNDE** | **F:5'-CGAGAATTCACGCGTGGTACCATGTTGGC-3'**  **R:5'-CGGGTCGACTCTAGAGGTACCTTATAGTC-3'** |
| **19** | **Lnc-HCP5** | **F:5'-CCGCTGGTCTCTGGACACATACT-3'**  **R:5'-CTCACCTGTCGTGGGATTTTGC-3'** |
| **20** | **Lnc-FENDRR** | **F:5'-CCACATGGATGGTTGCCACTCTC-3'**  **R:5'-GCTGGTACTCGGCCTTCTAATTGG-3'** |
| **21** | **CDKN2BAS** | **F:5'-TGCCGGAGCTGTCGACCC-3'**  **R:5'-TGATCTCTGCTGTTGAATCAGAATG-3'** |
| **22** | **MCM3AP-AS1** | **F:5'-GCTGCTAATGGCAACACTGA-3'**  **R:5'-AGGTGCTGTCTGGTGGAGAT-3'** |
| **23** | **miR-24-3p** | **F:5'-TGTGGGAACAGCTCTTCTCC-3'** |
|  |  | **R:5'-AAGATTCAAGTCAAAATTGT-3'** |
| **24** | **BCL2L2** | **F:5'-GCGGAGTTCACAGCTCTATAC-3'** |
|  |  | **R:5'-AAAAGGCCCCTACAGTTACCA-3'** |
| **25** | **IL1A** | **F:5'-TGGTAGTAGCAACCAACGGGA-3'** |
|  |  | **R:5'-ACTTTGATTGAGGGCGTCATTC-3'** |
| **26** | **GSK3B** | **F:5'-TGGTCGCCATCAAGAAAGTATTG-3'** |
|  |  | **R:5'-GCGTCTGTTTGGCTCGACTAT-3'** |
| **27** | **BCL2L11** | **F:5'-TAAGTTCTGAGTGTGACCGAGA -3'** |
|  |  | **R:5'-GCTCTGTCTGTAGGGAGGTAGG-3'** |
| **28** | **GAPDH** | **F:5'-GGAGCGAGATCCCTCCAAAAT-3'** |
|  |  | **R:5'-GGCTGTTGTCATACTTCTCATGG-3'** |
| **29** | **U6** | **F:5'-CTCGCTTCGGCAGCACA-3'** |
|  |  | **R:5'-AACGCTTCACGAATTTGCGT-3'** |
|  |  |  |
